# 3D printed mold leachates in PDMS microfluidic devices

**DOI:** 10.1038/s41598-020-57816-y

**Published:** 2020-01-22

**Authors:** Marcia de Almeida Monteiro Melo Ferraz, Jennifer Beth Nagashima, Bastien Venzac, Séverine Le Gac, Nucharin Songsasen

**Affiliations:** 1 Center for Species Survival, Smithsonian National Zoo and Conservation Biology Institute, 1500 Remount Road, Front Royal, Virginia 22630 USA; 2 Applied Microfluidics for Bioengineering Research, MESA+Institute for Nanotechnology and TechMed Center, University of Twente, 7500 Enschede, AE The Netherlands

**Keywords:** Lab-on-a-chip, Mass spectrometry

## Abstract

The introduction of poly(dimethylsiloxane) (PDMS) and soft lithography in the 90’s has revolutionized the field of microfluidics by almost eliminating the need for a clean-room environment for device fabrication. More recently, 3D printing has been introduced to fabricate molds for soft lithography, the only step for which a clean-room environment is still often necessary, to further support the rapid prototyping of PDMS microfluidic devices. However, toxicity of most of the commercial 3D printing resins has been established, and little is known regarding the potential for 3D printed molds to leak components into the PDMS that would, in turn, hamper cells and/or tissues cultured in the devices. In the present study, we investigated if 3D printed molds produced by stereolithography can leach components into PDMS, and compared 3D printed molds to their more conventional SU-8 counterparts. Different leachates were detected in aqueous solutions incubated in the resulting PDMS devices prepared from widely used PDMS pre-polymer:curing agent ratios (10:1, 15:1 and 20:1), and these leachates were identified as originating from resins and catalyst substances. Next, we explored the possibility to culture cells and tissues in these PDMS devices produced from 3D printed molds and after proper device washing and conditioning. Importantly, we demonstrated that the resulting PDMS devices supported physiological cultures of HeLa cells and ovarian tissues *in vitro*, with superior outcomes than static conventional cultures.

## Introduction

Organ-on-a-chip models, by providing an *in vivo*-like environment, show great potential to advance our understanding of tissue development, physiology, and pathology^1^. However, the spread of these highly promising platforms out of specialized microfluidic laboratories is hindered by essential technical challenges. The fabrication of these devices requires having access to dedicated infrastructure such as clean-room facilities with specialized equipment, which is absent in most biological laboratories^1^. Novel fabrication processes have been developed to support the rapid prototyping of microfluidic devices, including soft-lithography using PDMS (polydimethylsiloxane)^2,3^. PDMS is a transparent, flexible, gas-permeable, fairly inexpensive and rapidly prototyped elastomeric material, which has now become ubiquitous in the field of microfluidics^2,3^. PDMS devices are commonly fabricated using molds based on SU-8 (Microchem Corp.), which is a negative epoxy-based photoresist^4^. However, the fabrication of SU-8 molds uses lithography and includes multiple steps, it altogether takes several hours per fabrication cycle, and still requires dedicated training and access to a clean-room facility^5,6^.

As a new revolution in the field of microfluidics, 3D printing has more recently been introduced^7^. Initially the use of 3D printing was limited by the price of the printers, their low resolution, the roughness of the 3D printed structures, and the toxicity of the resins^8–10^. Photopolymerization-based techniques, such as digital light processing (DLP) and stereolithography (SLA) allow the fabrication of complex 3D structures with high resolution, which has expanded the use of 3D printing to several fields, including microfluidics, surgery, soft robotics, tissue engineering, drug delivery, dentistry, and the production of biomedical devices^7,10–12^. Nevertheless, the resin toxicity remains a significant issue. ﻿Resin constituents such as uncured monomers, short-chain polymers, photo-initiators, and other auxiliary compounds can leach from the printed parts in solution, which is often not compatible with biological applications^13^. For instance, Zhu *et al*. demonstrated that leachates from seven commercially available polymer resins resulted in significant growth inhibition of freshwater microalgae, and high (50–100%) mortality of several common aquatic toxicity bioassay species within 96 h exposure^14^. Moreover, 3D printed photopolymers were proven to be lethal to zebrafish embryos^9,15^. Finally, in previous work, we found that 3D printed parts released toxic compounds in solution, including phthalates and polyethylene glycol (PEG), which impaired bovine embryonic development *in vitro*^8^. A variety of strategies have been proposed to overcome the limitations of 3D printing for biological microfluidic applications, including post-curing washes with ethanol and coating of the printed devices^9,14,15^. For example, wax coating of 3D printed parts delayed toxicity to zebrafish embryos for *ca*. 40 h, which was attributed to the slower diffusion of toxic materials from the resin into the culture medium thanks to the coating^9^. Similarly, 3D printed devices were coated with PDMS and polystyrene before endothelial cell culture^16^.

Currently, 3D printed molds are increasingly used to cast PDMS devices as an alternative to SU-8 molds^10,17^. While this strategy removes the direct contact of 3D printed resins with biological materials, questions remain on the fate of leachates from the mold and their potential transfer into the PDMS matrix during curing to eventually end up in the cell/tissue culture compartment. In this study, we 1) compared, using mass spectrometry (MS) analysis, the leachate compounds from PDMS devices prepared from commonly used pre-polymer:curing agent ratios (10:1, 15:1, and 20:1) and cast using SLA-printed molds as well as conventional SU-8 molds; and 2) assessed the applicability of these PDMS devices fabricated using 3D printed molds for both HeLa cell monolayer and ovarian tissue cultures.

## Results and Discussion

### Both 3D-printed and SU-8 molds do leach into PDMS

A variety of compounds can be released from 3D printed molds^8,9,14,15^ and these compounds can have different properties, such as thermo-resistance. Therefore, different MS-based approaches must be combined for detecting all these leachates^18^. Gas chromatography-mass spectrum (GC-MS) primarily allows characterizing a vast number of volatile and small compounds^18^, such as photo-initiators, but highly polar compounds must be derivatized to increase volatility before analysis and high molecular weight compounds cannot be analyzed by GC-MS, which represents a significant limitation of this technique^19,20^. In contrast, liquid chromatography with tandem mass spectrometry (LC-MS/MS) is well-suited for analyzing higher molecular weight and non-volatile compounds, covering a wide range of polarities without the need for prior derivatization^19^. In the present study to ensure that all possible leachates were identified, we used a combination of these two techniques, GC-MS and LC-MS/MS.

Here, we considered different pre-polymer:curing agents ratios for the production of the PDMS devices. While a 10:1 ratio is standard in the microfluidics field, other ratios such as a 15:1 and 20:1 ratio are used to facilitate PDMS bonding^21^, for fabricating culture devices to study mechanobiology^22^, for the creation of flexible pressure sensors^23^, and to integrate valves and/or pumps^24,25^. Importantly, changes in the PDMS pre-polymer:curing agent ratio affects the porosity of the resulting PDMS material and increase the amount of free, non-crosslinked oligomers, which are known to segregate to the surface of the bulk PDMS^22^. The resulting coating can potentially change interactions of the PDMS with other substrates^22^, and in the present study with 3D printed parts.

First, leachates collected by incubating the PDMS devices (pre-polymer:curing agent ratios of 10:1, 15:1 and 20:1, fabricated form either 3D printed or SU-8 molds) with MilliQ water (1 μL of water per 1 mm^3^ of PDMS surface) for 24 h and analyzed by GC-MS. Although we could not determine their origin, several compounds were putatively identified, based on queries against NIST EI database, from devices produced using both 3D printed and SU-8 molds after GC-MS analysis (Table 1 and corresponding Supplementary Figs. 1–30), such as nitriles (fumaronitrile and 2-cyanosuccinonitrile), phosphorous [(chloromethyl)dimethyl-phosphine oxide], and sulfoxide [(2,3-diphenylcyclopropyl)methyl phenyl sulfoxide]. Succinonitriles, for instance, can be metabolized into cyanide both *in vivo* and in *in vitro* cultured liver slices^26^, suggesting that the potential effects of these leachates on biological samples should be investigated whenever the latter could be exposed to these compounds. GC-MS analysis also revealed the presence of specific constituents for PDMS devices fabricated from SU-8 molds: chalcone compounds, 2-chloro-ethanesulfonyl chloride and 4-methoxybenzyl alcohol. Chalcones are very reactive upon UV irradiation and are commonly used in the formulation of photoresists such as SU-8^27,28^. Despite their anti-inflammatory, anti-oxidant, anti-nociceptive, anti-parasites, and anti-proliferative ﻿pharmaceutical effects, chalcones have also been found to have a myotoxic effect in zebrafish^29^, and to stimulate apoptosis in human colorectal carcinoma cells^30^. 2-Chloro-ethanesulfonyl chloride, which is also part of photoresist solvents, is known to be corrosive and cause acute toxicity^31^.

**Table 1:** Putatively identified compounds (based on queries against NIST EI database) of GC-MS analyzed Milli-Q water (H_2_O) conditioned or not with PDMS devices prepared from various pre-polymer:curing agent ratios (20:1, 15:1, and 10:1) fabricated from either 3D-printed (3D) or SU-8 (SU-8) molds with their retention time (RT) and similarity score (SI).

Mold	Sample	RT (min)	SI*	Compound	Origin	m/z spectra
3D	10:01	4.216	447	N-Benzenesulfonylazetidin-3-one	Polyethylene	Supp. Fig. 1
		4.332	458	(2,3-Diphenylcyclopropyl)methyl phenyl sulfoxide	Unknown	Supp. Fig. 2
		4.582	434	4-Phenylbutan-2-ol, tert-butyldimethylsilyl ether	Unknown**	Supp. Fig. 3
		5.997	715	Fumaronitrile	Plastic monomer	Supp. Fig. 4
		10.176	636	1,1,3,3,5,5,7,7,9,9,11,11-Dodecamethyl-hexasiloxane	PDMS oligomer	Supp. Fig. 5
	15:01	6.214; 11.908; 28.392	548; 640;635	1,1,3,3,5,5,7,7,9,9,11,11-Dodecamethyl-hexasiloxane	PDMS oligomer	Supp. Fig. 6
		6.597	826	Toluene	PDMS curing agent	Supp. Fig. 7
		5.947	459	2-Cyanosuccinonitrile	Unknown	Supp. Fig. 8
		4.299	504	4-(4-chlorobenzylideno)-3-methyl-1-phenyl-pyrazol-5(4 H)-one	Unknown	Supp. Fig. 9
		4.166	409	2,2,2-Trichloroethanol, methyl ether	Unknown	Supp. Fig. 10
	20:01	6.214	615	1,1,3,3,5,5,7,7,9,9,11,11-Dodecamethylhexasiloxane	PDMS oligomer	Supp. Fig. 11
		15.887; 24.446	702; 615	Dodecamethylcyclohexasiloxane	PDMS oligomer	Supp. Fig. 12
		11.192	800	Octamethyltetrasiloxane	PDMS oligomer	Supp. Fig. 13
		10.143	632	Methyl N-hydroxybenzenecarboximidoate	Fungicide**	Supp. Fig. 14
		7.179	933	Dimethylsilanediol	Degradation of silicones**	Supp. Fig. 15
SU-8	10:01	12.341	660	1,1,3,3,5,5,7,7,9,9,11,11,13,13,15,15-Hexadecamethyl-octasiloxane	PDMS oligomer	Supp. Fig. 16
		5.731	529	4-Methyl-2-trimethylsilyloxy-trimethylsilyl ester benzoic acid	Unknown	Supp. Fig. 17
		4.332	419	(2-Benzyl-benzoimidazol-1-yl)-propane-1,2-diol	Unknown	Supp. Fig. 18
		4.099	410	Pyrazol-5(4 H)-one, 4-(4-chlorobenzylideno)-3-methyl-1-phenyl	Unknown	Supp. Fig. 19
	15:01	29.574	673	1,1,3,3,5,5,7,7,9,9,11,11-Dodecamethyl-hexasiloxane	PDMS oligomer	Supp. Fig. 20
		8.944	671	Octamethyl-cyclotetrasiloxane	PDMS oligomer	Supp. Fig. 21
		5.514	741	4-Methoxybenzyl alcohol	Catalyst	Supp. Fig. 22
		5.365	594	4,6-Heptadiyn-3-one	Unknown	Supp. Fig. 23
		4.416	510	2-(Chloroethenyl)-1,3-butadiene	Unknown	Supp. Fig. 24
		4.266	573	(Chloromethyl)dimethyl-phosphine oxide	Unknown	Supp. Fig. 25
		4.149	573	2-Chloro-ethanesulfonyl chloride	Epoxy resin component	Supp. Fig. 26
	20:01	13.457	676	1,1,3,3,5,5,7,7,9,9,11,11-Dodecamethyl-hexasiloxane	PDMS oligomer	Supp. Fig. 27
		6.014	690	1,1,3,3,5,5,7,7-Octamethyl-7-(2-methylpropoxy)tetrasiloxan-1-ol	PDMS oligomer	Supp. Fig. 28
		4.449	525	Chalcone	Epoxy resin component	Supp. Fig. 29
		4.366	454	4-(4-Chlorobenzylideno)-3-methyl-1-phenyl-pyrazol-5(4 H)-one	Unknown	Supp. Fig. 30

*SI was calculated based on EI peak matching of the experimental data against the NIST EI database.

**indicates compounds also detected in water (control samples).

**Figure 1:**
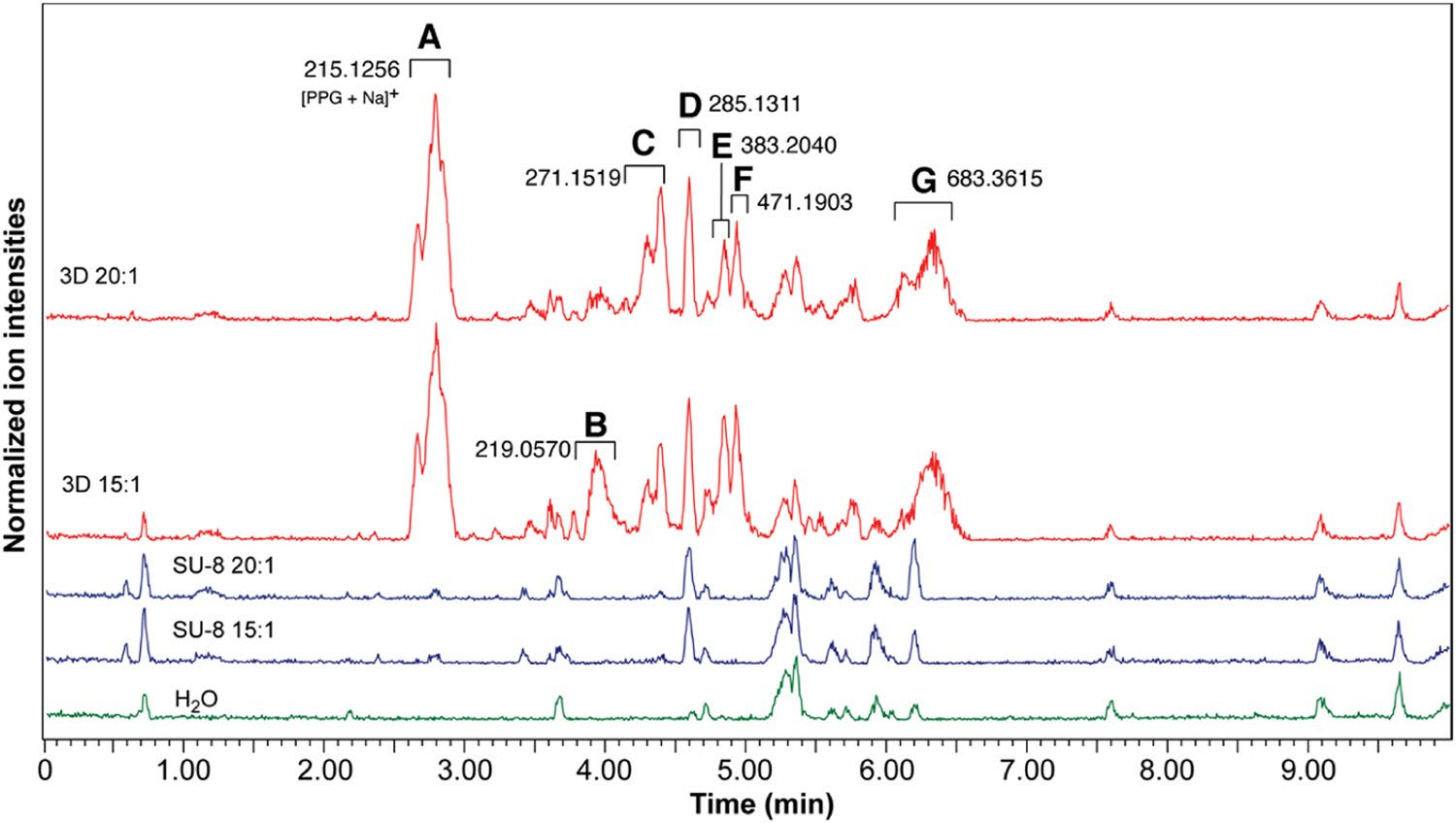
LC-MS/MS base peak ion chromatograms (positive ion mode) of Milli-Q water samples incubated in various PDMS devices or not (control sample). Devices were fabricated from 3D printed (3D) or SU-8-based molds (SU-8), using PDMS pre-polymer:curing agent ratios of 15:1 and 20:1. Monoisotopic mass values are provided for the ions that differ across the samples.

Next, LC-MS/MS analysis was performed, notably for comparing PDMS devices prepared from 3D printed and SU-8 molds, and using pre-polymer:curing agent ratios of 20:1 and 15:1. Representative base peak ion chromatogram (of positive ion mode) for these analyses are presented in Fig. 1. Detected species highlighted with a letter in Fig. 1, were isolated and subjected to MS/MS analysis in an attempt to derive their structure and, except for peak A, no conclusive results were found. Aqueous samples in contact with PDMS devices fabricated from 3D printed molds gave rise to 6 unique peaks (A, B, C, E, F and G) that were not identified in the control (H_2_O) and SU-8 samples. Peak D was identified for both 3D printed and SU-8 samples. Peak A corresponds to polypropylene glycol (PPG) as a sodium adduct and was the mostly abundant leachate present. PPG is an aliphatic alcohol that is vastly used in cosmetics, as food additive and as vehicle for many drugs^32,33^. Moreover, PPG is also a component of resins used for stereolithography printing^34^. Although PPG is a “generally recognized as safe” additive for foods and medications^33^, it was shown to be toxic to human proximal tubule cells^35,36^. Therefore, PPG possible toxic effects on *in vitro* cell cultures should be investigated before using PDMS devices produced by 3D printed molds. Altogether, leachates from molds are transferred into the PDMS porous matrix, from which they are subsequently released into the solution introduced in the microfluidic device. Importantly, devices produced from either SU-8 or 3D printed molds resulted in leachates with concerning potential toxicities.

### PDMS devices fabricated using 3D printed molds support physiological cell/tissue growth *in vitro*

**Figure 2:**
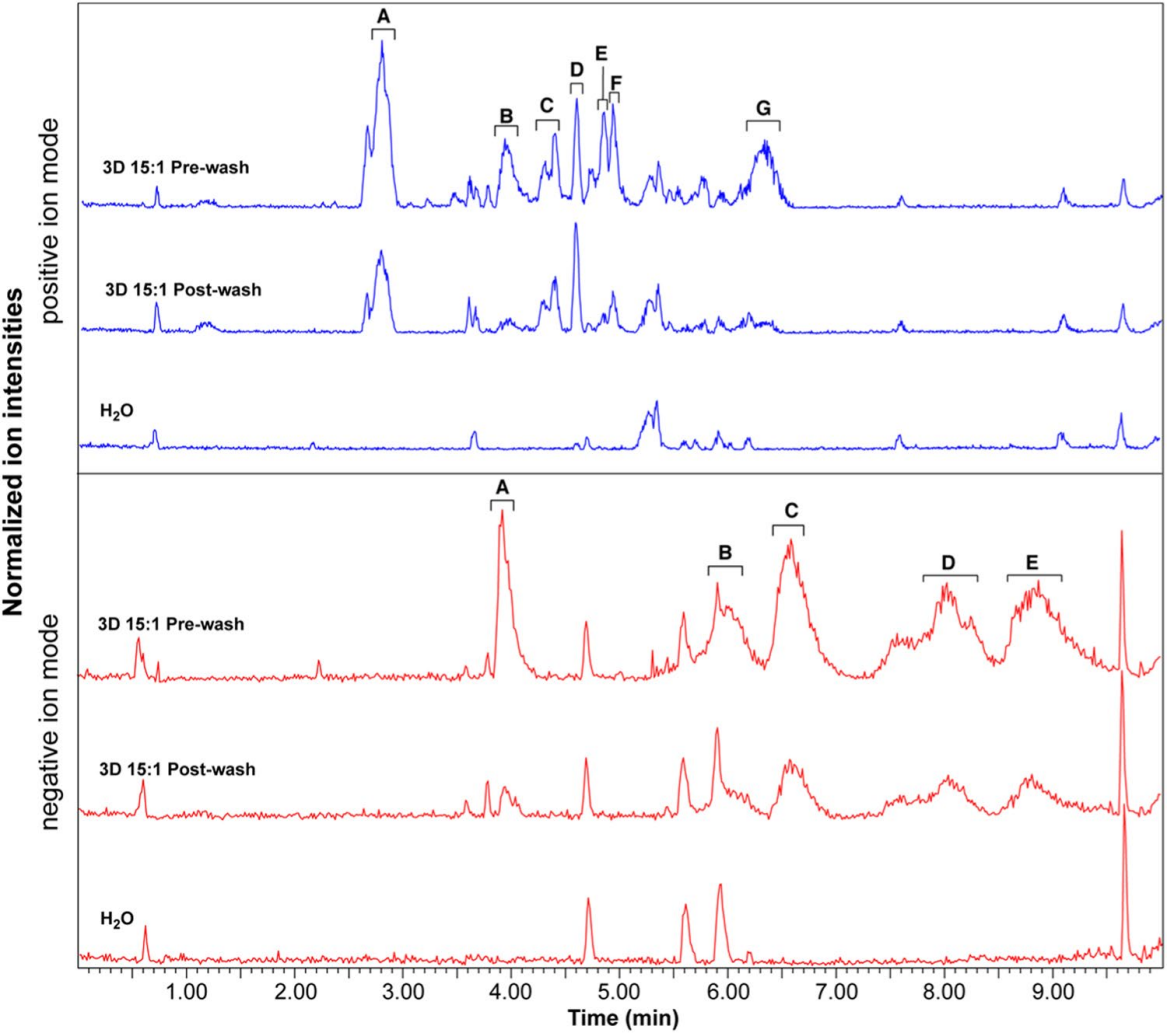
The effect of washing on leachates. LC-MS/MS base peak ion chromatograms of Milli-Q water conditioned or not (H_2_O) with PDMS devices fabricated from 3D printed molds (pre-polymer:curing agent ratio of 15:1), with (Post-wash) and without (Pre-wash) an overnight wash. Both positive and negative ion base peak chromatograms are shown. Identified peaks correspond to the main ions that differ across the samples.

The potential adverse effects of culturing cells in PDMS devices was previously described^37–39^. Although components of the culture medium can absorb in the porous polydimethylsiloxane matrix and their concentrations changed in solution^40,41^, we previously demonstrated that absorption of hormones from the perfusion medium happens within 12–24 h in PDMS chips, and no extra absorption or release of hormones were observed after this period^42^. To avoid the negative effects of reduced concentration of medium supplements (such as the Follicle Stimulating Hormone), we conditioned the PDMS devices overnight previously to cell and tissue cultures with supplemented culture medium. To investigate if this overnight conditioning step would allow removing the mold leachates, we incubated our culture devices (15:1 pre-polymer:curing agent ratio, fabricated from 3D printed molds) for 24 h with MilliQ water (pre-wash sample) and conditioned devices for an extra 24 h (post-wash sample). Pre- and post-wash samples were analyzed by LC-MS/MS and GC-MS as before. Although peaks were still detected in the post-wash samples, their surface area was reduced, as seen in the LC-MS/MS base peak chromatograms (Fig. 2). With exception of peaks C and D from the positive ion mode, all peaks in post-wash samples had more than 50% reduction in their surface area. The putatively identified compounds by GC-MS in post-washed devices were mostly of PDMS origin or contaminants also detected in control water samples (Supplementary Table 1 and corresponding MS spectra Supplementary Figs. 32–35).

We next sought to culture cell lines and tissues in the devices fabricated using 3D printed molds, as a proof-of-concept. For cell culture, a device containing a square chamber (3 mm long, 3 mm wide, and 1 mm high), linked to two inlet and outlet channels (2 mm long, 1 mm wide, and 1 mm high) by a funnel section, having identical bottom and top parts, with an intermediate porous membrane was used (Supplementary Fig. S36a). While tissue culture devices also comprised two compartments, the top compartment was a single elongated channel (24 mm long, 3 mm wide, and 4 mm high), and the bottom compartment was identical to the top compartment but smaller in height (1 mm; Supplementary Fig. S36b). These devices were produced using a 15:1 prepolymer:curing agent ratio, which is better suited for easy assembly of the devices without any need for specialized equipment.

**Figure 3:**
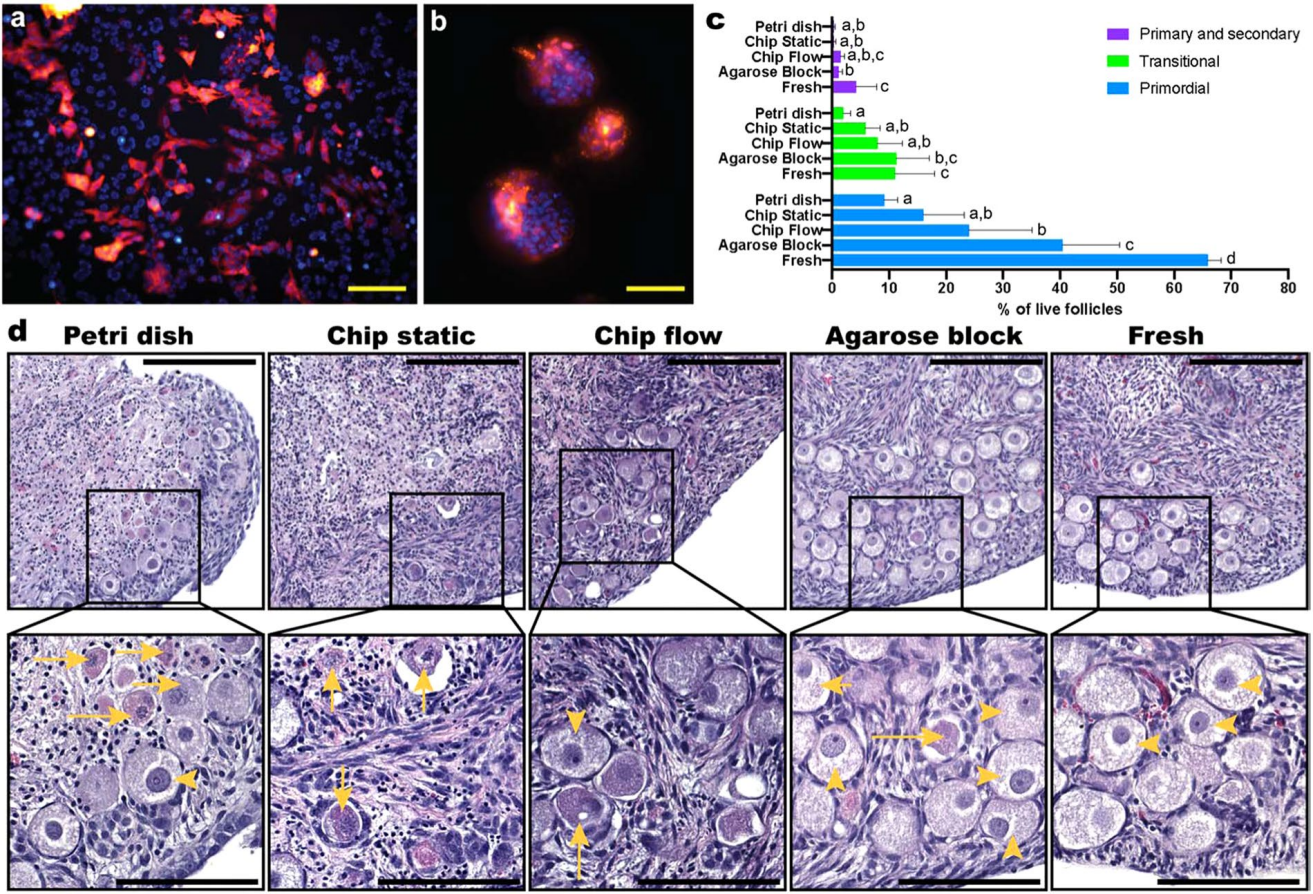
Cell and *ex vivo* tissue culture in the Organ-on-a-chip platforms. RFP-labelled Hela cells were cultured for 4 days under perfusion in the cell culture device, displaying (**a**) a confluent monolayer and normal morphology in the culture chamber, and (**b**) spheroids/spherical aggregates in the inlet and outlet microchannels (red RFP; blue – nuclei stained with HOECHST3342). Ovarian cortical tissues from four 9- and 10-week old domestic cats were cultured for 4 days in the tissue culture device: (**c**) percentages of live primordial, transitional, and primary and secondary stage follicles from each treatment group, of which representative images are displayed for (**d**) tissue samples cultured submerged in a petri dish, in the microfluidic devices under static and flow conditions, on agarose block, and freshly collected tissues. Top scale bars (yellow) represent 200 µm and bottom ones (black) 100 µm; yellow arrowheads indicate morphologically normal, live primordial follicles, and entire arrows atretic follicle.

In a first experiment, RFP-expressing HeLa cells were seeded in the top fluidic compartment and cultured under perfusion (0.5 µl min^−1^ flow in both the top and bottom compartments) for 4 days. HeLa cells presented healthy morphology throughout the culture in the device (Fig. 3a). Interestingly, cells formed spheroid-like structures in the microchannels close to the inlets, where the shear stress was significantly higher (0.429 *vs*. 0.016 dyne cm^−2^ in the culture chamber) (Fig. 3b), much akin the structures obtained when culturing HeLa cells under 3D conditions^43^. Importantly, spheroid formation has been previously described when cells were exposed to shear stress^44^, as is the case in our study. Therefore, we hypothesize that spheroid formation was a response to the higher shear stress HeLa cells experienced in the channels, which illustrates normal physiological response of these cells in our device.

Next, domestic cat ovarian cortical tissues were cultured under four different conditions: static in a petri dish, the tissue piece being submerged in culture medium; static on agarose gel, the tissues being placed on top of an agarose gel block which was partially submerged in culture medium, and exposed to an air-liquid interface^45^; static on chip; and dynamic on-chip culture with a 2 µl min^−1^ continuous flow applied in the bottom chamber. In all ‘static’ conditions, culture medium was refreshed every second day. Mammalian ovaries contain hundreds of thousands of immature or “primordial” follicles, comprising granulosa cells and the enclosed oocytes^46^. The majority of the oocytes from these primordial follicles will never be ovulated and the ability to rescue and grow these follicles *in vitro* is of extreme importance to rescue female fertility^46^. Ovarian tissues are sensitive to culture; specifically, nutrient availability is a problem for their survival and a significant reduction in follicle viability has been reported/observed when these tissues are statically cultured^45^. Here, the proportion of morphologically normal live follicles was maintained in the microfluidic device after four days of dynamic culture compared with the traditional tissue culture on an agarose block (40.84 ± 15.02 and 52.80 ± 15.49%, respectively; p = 0.5369), which was also higher than for tissues cultured under static conditions in a petri dish (11.27 ± 3.89%; p = 0.0013 *vs*. dynamic on-chip and p < 0.001 *vs*. agarose block). Nevertheless, all culture conditions had reduced number of live follicles when compared to the fresh tissue (81.06 ± 4.97%; vs agarose block: p = 0.00269, vs dynamic on-chip: p < 0.001, vs static on-chip: p < 0.001 and vs petri dish: p < 0.001). As seen in Fig. 3c and the representative pictures in Fig. 3d, tissues cultured in the PDMS devices under flow conditions had a higher number of primordial follicles than tissues cultured in the petri dish (p = 0.0433). Moreover, tissues cultured in the PDMS devices under flow conditions had similar number of transitional and primary and secondary follicles as fresh tissues (p = 0.8517 and 0.09611, respectively) and tissues cultured on an agarose block (p = 0.7989 and 0.9982, respectively), collectively demonstrating that the PDMS devices fabricated from 3D printed molds had no adverse effect on ovarian tissue culture. Altogether, these results demonstrate that the leachates from 3D printed molds into PDMS are partially removed after overnight conditioning and that PDMS devices manufactured from 3D printed molds are suitable for physiological growth of HeLa cells and ovarian tissue survival in short-term *in vitro* cultures.

## Conclusion

The use of 3D printed molds to rapidly prototype and fabricate microfluidic devices has increased in the past years, which facilitated and supported the expansion of this technology to non-specialized laboratories. Although the biocompatibility of 3D printed resins has been investigated, the studies were focused on whether or not 3D printed resins leachates when in direct contact with aqueous solutions or solvents. Here, we demonstrated that leachates are present in solutions incubated in PDMS devices fabricated using 3D printed molds, despite SLA resins not coming into direct contact with the aqueous solutions. Nevertheless, PDMS devices fabricated from 3D printed molds supported viable culture of HeLa cells and *ex vivo* ovarian tissues. In sum, 3D printing is a useful alternative to the laborious clean-room produced SU-8 molds for fabricating multilayer PDMS devices. However, caution should be taken in evaluating the potential toxicity of resin-based leachates in PDMS devices fabricated from 3D printed molds and implementing suitable device preparation and conditioning steps to eliminate any risk for toxicity.

## Material and Methods

### Device design and fabrication

Microfluidic devices were designed using SolidWorks (Dassault Systems, Velizy-Villacoublay, France); they comprised two compartments separated by a 10 µm-thick polycarbonate porous membrane. Both compartments consisted of a single elongated channel (24 mm long and 3 mm wide) and had respective heights of 4 mm and 1 mm, for the top and bottom compartments. Molds were printed using an SLA FlashForge Hunter printer (Jinhua, China) using Fun-To-Do Industrial Blend resin (Alkmaar, The Netherlands), and post-cured under 405 nm UV light at 14 mW/cm^2^ for 2 h followed by 24 h at 60 °C in order to avoid curing inhibition of the PDMS by 3D printed mold leachate^17^. In parallel, SU-8 molds were also produced to fabricate “control” PDMS devices for the leachate analysis experiments. Those devices containing a channel (30 mm long, 3 mm wide and 300 µm high) were designed using Clewin software (WieWeb, The Netherlands). The SU-8 based molds were produced using SU8–100 resin (Microchem) according to the manufacturer’s instructions in the NanoLab cleanrooms of the MESA + Institute for Nanotechnology.

PDMS mixtures (Sylgard 184, Dow Corning Midland, United States) with pre-polymer:curing agent ratios of 20:1, 15:1, and 10:1 were poured on the 3D printed molds or SU-8 molds and partially cured for 30, 25 and 20 min at 62 °C in an oven, respectively. The top and bottom compartments were peeled off the molds, and horizontal inlets and outlets were created using a 1.5-mm biopsy punch (Integra® Miltex®, USA). The top and bottom layers were aligned with a polycarbonate porous membrane in the middle (Nuclepore^™^ Track-Etched Membranes, pore size: 0.4 µm, Whatman^®^, USA), then totally cured at 62 °C overnight, with a weight on top to ensure both parts were properly assembled. After curing, silicone tubes (o.d. 1.5 mm, i.d. 0.5 mm, Tygon tubing, Cole-Parmer^®^, USA) were secured in the inlets and outlets using PDMS glue (10:1 pre-polymer:curing agent ratio), which was cured for 30 min at 62 °C.

Devices produced using a polymer:curing agent ratios of 15:1 were used for tissue culture after a 5 mm × 3 mm opening was made into the top compartment with a scalpel for later tissue insertion, which was closed with pressure-sensitive adhesive tape (plate sealer tape, Thermo Scientific, USA)^47^. Another device, containing a square chamber (3 mm long, 3 mm wide, and 1 mm high), linked to two inlet and outlet channels (2 mm long, 1 mm wide, and 1 mm high) by a funnel section, having identical bottom and top parts, with an intermediate porous membrane, was designed and fabricated as described above using a polymer:curing agent ratios of 15:1 and used for cell culture. Before use for culture, the devices were washed with 1 mL of a 70% ethanol aqueous solution, followed by 3 washes with 1 mL of Milli-Q water, UV-sterilized for 30 min, and then perfused overnight in DMEM/F12 medium (Gibco, USA) supplemented with 100 U ml^−1^ penicillin and 100 µg ml^−1^ streptomycin (Gibco, USA) at a 2 μL min^−1^ flow-rate.

The shear stress in the chamber and the channel of the cell culture device was calculated using the online tool provided by Darwin microfluidics (https://darwin-microfluidics.com/blogs/tools/microfluidic-flowrate-and-shear-stress-calculator).

### GC-MS and LC-MS/MS analysis

After assembly, devices were washed 3 times with Milli-Q water, UV sterilized for 30 min and filled with Milli-Q water. For both 3D printed and SU-8 casted devices, 1 μL of water per 1 mm^3^ of chamber surface was used for incubation. Three devices from each 3D printed and SU-8 molds (10:1, 15:1 and 20:1, n = 18) were incubated with water (288 and 27 μL for 3D printed and SU-8 PDMS casted devices, respectively) for 24 h (37 °C, 5% CO_2_ in a humidified incubator), after which the water from the 3 devices was pooled and frozen at −20 °C until analysis. Milli-Q water incubated in a sterile polystyrene 1.5 ml centrifuge tube was used as a control. After a first water incubation step and collection (pre-wash samples), 15:1 devices fabricated from 3D printed molds (n = 3) were filled with Milli-Q water, incubated for 24 h (37 °C, 5% CO_2_ in a humidified incubator), after which the water from the 3 devices were pooled and frozen at −20 °C until analysis (post-wash samples). Samples were sent to the mass spectrometry facility of the Department of Chemistry and Biochemistry of the University of Maryland (MD, USA), for analysis by GC-MS (gas chromatography coupled to mass spectrometry) and to the mass spectrometry facility of the Department of Biochemistry of the Virginia Tech University for LC-MS/MS analysis.

GC-MS measurements were performed on an Agilent 6890 N system coupled with a JEOL high-resolution magnetic sector mass spectrometer (JMS-700 MStation) equipped with an EI (electron ionization) source (70 eV). The mass spectrometer was operated in the high scan speed and low resolution (1000) mode, for a mass range of 50–500 Da. A silica capillary column (Agilent DB-5, 60 m length, 250 μm I.D.) was used with helium (at 1 ml min^−1^) as the carrier gas. Analysis was performed as follows: injection volume 0.2 μL; inlet temperature 270 °C in spitless mode; column temperature set at 50 °C at 5.0 min, next increased to 300 °C at the rate of 12 °C min^−1^ and finally held at 300 °C for another 4.2 min. Data acquired by GC-MS were further analyzed using JEOL Work Manager software (version 1.3) and the NIST MS dataset (version 2.0, 2011) to identify the compounds corresponding to each experimental spectrum detected. These compounds are listed in Table 1 with the associated similarity scores (SI), indicating the certainty of it being the said compound. The SI was calculated based on the EI peak matching of the experimental data against the NIST EI database. Here, data analysis only yielded one possible compound per detected species. Compound origin was based on research and patent literature search.

LC-MS/MS samples were prepared by supplementing the collected water (90 μL) with acetonitrile containing 0.1% formic acid (10 μL). Aliquots (10 μL) were injected onto a Waters BEH C18 column (2.1 mm × 50 mm, 1.7 μm particles) maintained at 35 °C. A binary gradient was used with solvent A, 0.1% formic acid in water, and solvent B, 0.1% formic acid in acetonitrile. The elution gradient was as follows: 0–0.5 min 5% B, 0.5–8 min linear ramp from 5–90% B, 8–8.5 min hold a 90% B, 9 min return to initial conditions. Column eluents were analyzed on-line using electrospray ionization-mass spectrometry with a Waters Synapt G2S hybrid Q-ToF. The source parameters were the same for the positive and negative modes with the exception of the capillary voltage which was 3.0 kV in positive mode and 2.4 V in negative mode (source temperature 120 °C, sampling cone 30 V, source offset 80, desolvation temperature 350 °C, cone gas 50 L h^−1^, desolvation gas 500 L h^−1^, and nebulizer gas 6 bar). Data were collected from 50–1,800 *m/z* in MS^E^ mode with a scan time of 0.1 s and mass correction was performed with leucine enkephalin infused through the lock spray source and analyzed every 20 s.

### HeLa cell culture and imaging

RFP-expressing HeLa cells (GenTarget Inc, USA) were seeded in the top compartment of the devices and cultured under perfusion for 4 days (0.5 µl min^−1^ flow applied using a syringe-pump) with DMEM/F12 medium (Gibco, USA) supplemented with 10% fetal bovine serum (Sigma-Aldrich, USA), 2 mM L-glutamine (Sigma-Aldrich, USA), 0.1 mM MEM non-essential amino acids (Sigma-Aldrich, USA), 100 U ml^−1^ penicillin and 100 µg ml^−1^ streptomycin (Gibco, USA), at 37 °C, 5% CO_2_ in a humidified incubator. The first 24 h only the bottom chamber was perfused to allow cell attachment in the top chamber, and after one day both the bottom and top chambers were perfused at the same flow-rate (0.5 µl min^−1^). At day 4 microfluidic devices were washed three times with phosphate buffer saline (PBS, GIBCO, USA), cells were fixed with 4% paraformaldehyde for 30 min and stained with HOECHST33342 (5 μg mL^−1^, Invitrogen, USA) for 15 min. Devices were next opened, the porous membranes removed, placed on a glass slide with a glass coverslip, and cells imaged by fluorescence microscopy (EVOS FL auto 2, Invitrogen, USA), at a 200× magnification.

### Ovarian tissue collection, culture and analysis

All female reproductive tracts were opportunistically collected from local veterinary clinics after routine spaying procedures of household and stray cats. No additional permissions were required since these biological materials were designated for disposal via incineration.

Domestic cat ovarian cortical tissues (n = 4 individuals, aged 9–10 weeks) were collected within six hours post-routine ovariohysterectomy at a local veterinary clinic and transported to the laboratory in L-15 medium (Sigma-Aldrich, USA) supplemented with 80.5 µM penicillin G, 41.2 µM streptomycin sulfate, and 50.0 µM ascorbic acid. Tissues were cut into ~2 × 2 × 1 mm^3^ square sections and two pieces were placed in the upper chamber of the device for each animal, and cultured in either static (bottom channel filled with culture medium, which was refreshed after 48 hours of culture), or flow conditions (2 µl min^−1^ continuous flow through the bottom chamber). Culture medium, as previously utilized, consisted of Minimum essential medium (Sigma-Aldrich, USA) supplemented with 4.2 µg ml^−1^ insulin, 3.8 µg ml^−1^ transferrin, 5 ng ml^−1^ selenium, 2 mM L-glutamine, 100 µg ml^−1^ penicillin G sodium and streptomycin sulfate, 0.05 mM ascorbic acid, 1 mg ml^−1^ polyvinyl alcohol, and 10 ng ml^−1^ follicle stimulating hormone (FSH, porcine-derived, Vetoquinol, USA). The open top of the devices was sealed using PCR plate sealer (Thermo Scientific, USA) to prevent leakage after tissue insertion^47^. Tissues (two pieces/individual) were also statically cultured outside the microfluidics devices, either submerged in 500 µl culture medium in a 4-well petri dish or incubated on top of a 1.5% agarose gel block which was partially submerged in culture medium, as previously utilized for domestic cat ovarian tissue culture^45^. Tissues were cultured for four days, after which the device was disassembled by removing the sealing tape, and ovarian tissues were fixed in 4% paraformaldehyde for evaluation of follicle density and morphology compared with fresh tissues that were fixed at the time of initial tissue collection from the same individual. Morphologically normal follicle counts were determined in 10 sections of a 300-µm thick tissue, as previously described^46^. The percentage of live follicles (number of morphologically normal primordial, transitional, primary and secondary follicles, divided by the total number of live and atretic follicles, standardized per mm^2^ tissue), was compared among treatment groups.

Primordial stage follicles were defined as a centralized oocyte surrounded by a single layer of flattened granulosa cells. Transitional stages were observed to have a combination of flattened and cuboidal granulosa cells in a single layer, with primary follicles containing a single layer of cuboidal granulosa, and secondary stage follicles with at least two layers of granulosa cells. Representative images of each follicle stage are presented in Supplementary Fig. 31. Only follicles with visible nuclei were counted, and follicles with pyknotic nuclei were considered non-normal morphologically speaking or “atretic”.

### Statistical analysis

Data were analyzed using the R package nlme (ver. 3.1–141)^48^. Data are presented as mean ± standard deviation. Normality of the residual of treatments was checked by plotting the density, and all data proved to be normal. Effects of culture conditions on the percentage of live, primordial, transitional and primary and secondary follicles (dependent variables) were analyzed by a Linear Mixed-effects Model (LME) with a Tukey post-hoc, where culture conditions (fresh, petri dish, chip static, chip flow or agarose block) were the fixed factor, and individual cat the random effect.

## Supplementary information

Supplementary files.

## Data Availability

All necessary data are present in the manuscript and Supplementary file.
