# Supplementary material for: 3D printed mold leachates in PDMS microfluidic devices
Source: Sci Rep. 2020 Jan 22;10:994. doi: 10.1038/s41598-020-57816-y (PMC6976631; doi:10.1038/s41598-020-57816-y)
Supplement: Supplementary file 1 — Supplementary files. [file 41598_2020_57816_MOESM1_ESM.docx]

**3D printed mold leachates in PDMS microfluidic devices**

Marcia de Almeida Monteiro Melo Ferraz*^a^, Jennifer Beth Nagashima^a^, Bastien Venzac^b^, Séverine Le Gac^b^, and Nucharin Songsasen^a^

*^a^Center for Species Survival, Smithsonian National Zoo and Conservation Biology Institute, 1500 Remount Road, Front Royal, Virginia 22630, USA.*

*﻿^b^Applied Microfluidics for Bioengineering Research, MESA+ Institute for Nanotechnology and TechMed Center, University of Twente, 7500 AE Enschede, The Netherlands*

Corresponding Author

* E-mail: *ferrazm@si.edu*

**Supplementary files**

Supplementary Fig. 1. m/z spectra of GC-MS data from Milli-Q water (H2O) conditioned with 10:1 PDMS chips fabricated from 3D-printed (3D) molds with 4.216 retention time (RT). Top image is the experimental spectra, bottom image was acquired from the NIST library and middle image is a combination of both library and experimental spectra.

Supplementary Fig. 2. m/z spectra of GC-MS data from Milli-Q water (H2O) conditioned with 10:1 PDMS chips fabricated from 3D-printed (3D) molds with 4.332 retention time (RT). Top image is the experimental spectra, bottom image was acquired from the NIST library and middle image is a combination of both library and experimental spectra.

Supplementary Fig. 3. m/z spectra of GC-MS data from Milli-Q water (H2O) conditioned with 10:1 PDMS chips fabricated from 3D-printed (3D) molds with 4.582retention time (RT). Top image is the experimental spectra, bottom image was acquired from the NIST library and middle image is a combination of both library and experimental spectra.

Supplementary Fig. 4. m/z spectra of GC-MS data from Milli-Q water (H2O) conditioned with 10:1 PDMS chips fabricated from 3D-printed (3D) molds with 5.997 retention time (RT). Top image is the experimental spectra, bottom image was acquired from the NIST library and middle image is a combination of both library and experimental spectra.

Supplementary Fig. 5. m/z spectra of GC-MS data from Milli-Q water (H2O) conditioned with 15:1 PDMS chips fabricated from 3D-printed (3D) molds with 10.176 retention time (RT). Top image is the experimental spectra, bottom image was acquired from the NIST library and middle image is a combination of both library and experimental spectra.

Supplementary Fig. 6. m/z spectra of GC-MS data from Milli-Q water (H2O) conditioned with 15:1 PDMS chips fabricated from 3D-printed (3D) molds with 28.392 retention time (RT). Top image is the experimental spectra, bottom image was acquired from the NIST library and middle image is a combination of both library and experimental spectra.

Supplementary Fig. 7. m/z spectra of GC-MS data from Milli-Q water (H2O) conditioned with 15:1 PDMS chips fabricated from 3D-printed (3D) molds with 6.597 retention time (RT). Top image is the experimental spectra, bottom image was acquired from the NIST library and middle image is a combination of both library and experimental spectra.

******Supplementary Fig. 8. m/z spectra of GC-MS data from Milli-Q water (H2O) conditioned with 15:1 PDMS chips fabricated from 3D-printed (3D) molds with 5.947 retention time (RT). Top image is the experimental spectra, bottom image was acquired from the NIST library and middle image is a combination of both library and experimental spectra.

Supplementary Fig. 9. m/z spectra of GC-MS data from Milli-Q water (H2O) conditioned with 15:1 PDMS chips fabricated from 3D-printed (3D) molds with 4.299 retention time (RT). Top image is the experimental spectra, bottom image was acquired from the NIST library and middle image is a combination of both library and experimental spectra.

Supplementary Fig. 10. m/z spectra of GC-MS data from Milli-Q water (H2O) conditioned with 15:1 PDMS chips fabricated from 3D-printed (3D) molds with 4.166 retention time (RT). Top image is the experimental spectra, bottom image was acquired from the NIST library and middle image is a combination of both library and experimental spectra.

Supplementary Fig. 11. m/z spectra of GC-MS data from Milli-Q water (H2O) conditioned with 20:1 PDMS chips fabricated from 3D-printed (3D) molds with 24.446 retention time (RT). Top image is the experimental spectra, bottom image was acquired from the NIST library and middle image is a combination of both library and experimental spectra.

Supplementary Fig. 12. m/z spectra of GC-MS data from Milli-Q water (H2O) conditioned with 20:1 PDMS chips fabricated from 3D-printed (3D) molds with 15.887 retention time (RT). Top image is the experimental spectra, bottom image was acquired from the NIST library and middle image is a combination of both library and experimental spectra.

Supplementary Fig. 13. m/z spectra of GC-MS data from Milli-Q water (H2O) conditioned with 20:1 PDMS chips fabricated from 3D-printed (3D) molds with 11.192 retention time (RT). Top image is the experimental spectra, bottom image was acquired from the NIST library and middle image is a combination of both library and experimental spectra.

Supplementary Fig. 14. m/z spectra of GC-MS data from Milli-Q water (H2O) conditioned with 20:1 PDMS chips fabricated from 3D-printed (3D) molds with 10.143 retention time (RT). Top image is the experimental spectra, bottom image was acquired from the NIST library and middle image is a combination of both library and experimental spectra.

Supplementary Fig. 15. m/z spectra of GC-MS data from Milli-Q water (H2O) conditioned with 20:1 PDMS chips fabricated from 3D-printed (3D) molds with 7.179 retention time (RT). Top image is the experimental spectra, bottom image was acquired from the NIST library and middle image is a combination of both library and experimental spectra.

Supplementary Fig. 16. m/z spectra of GC-MS data from Milli-Q water (H2O) conditioned with 10:1 PDMS chips fabricated from SU-8 (SU-8) molds with 12.341 retention time (RT). Top image is the experimental spectra, bottom image was acquired from the NIST library and middle image is a combination of both library and experimental spectra.

******Supplementary Fig. 17. m/z spectra of GC-MS data from Milli-Q water (H2O) conditioned with 10:1 PDMS chips fabricated from SU-8 (SU-8) molds with 5.731 retention time (RT). Top image is the experimental spectra, bottom image was acquired from the NIST library and middle image is a combination of both library and experimental spectra.

******

Supplementary Fig. 18. m/z spectra of GC-MS data from Milli-Q water (H2O) conditioned with 10:1 PDMS chips fabricated from SU-8 (SU-8) molds with 4.332 retention time (RT). Top image is the experimental spectra, bottom image was acquired from the NIST library and middle image is a combination of both library and experimental spectra.

Supplementary Fig. 19. m/z spectra of GC-MS data from Milli-Q water (H2O) conditioned with 10:1 PDMS chips fabricated from SU-8 (SU-8) molds with 4.099 retention time (RT). Top image is the experimental spectra, bottom image was acquired from the NIST library and middle image is a combination of both library and experimental spectra.

Supplementary Fig. 20. m/z spectra of GC-MS data from Milli-Q water (H2O) conditioned with 15:1 PDMS chips fabricated from SU-8 (SU-8) molds with 29.574 retention time (RT). Top image is the experimental spectra, bottom image was acquired from the NIST library and middle image is a combination of both library and experimental spectra.

Supplementary Fig. 21. m/z spectra of GC-MS data from Milli-Q water (H2O) conditioned with 15:1 PDMS chips fabricated from SU-8 (SU-8) molds with 8.944 retention time (RT). Top image is the experimental spectra, bottom image was acquired from the NIST library and middle image is a combination of both library and experimental spectra.

Supplementary Fig. 22. m/z spectra of GC-MS data from Milli-Q water (H2O) conditioned with 15:1 PDMS chips fabricated from SU-8 (SU-8) molds with 5.514 retention time (RT). Top image is the experimental spectra, bottom image was acquired from the NIST library and middle image is a combination of both library and experimental spectra.

******Supplementary Fig. 23. m/z spectra of GC-MS data from Milli-Q water (H2O) conditioned with 15:1 PDMS chips fabricated from SU-8 (SU-8) molds with 5.365 retention time (RT). Top image is the experimental spectra, bottom image was acquired from the NIST library and middle image is a combination of both library and experimental spectra.

Supplementary Fig. 24. m/z spectra of GC-MS data from Milli-Q water (H2O) conditioned with 15:1 PDMS chips fabricated from SU-8 (SU-8) molds with 4.416 retention time (RT). Top image is the experimental spectra, bottom image was acquired from the NIST library and middle image is a combination of both library and experimental spectra.

Supplementary Fig. 25. m/z spectra of GC-MS data from Milli-Q water (H2O) conditioned with 15:1 PDMS chips fabricated from SU-8 (SU-8) molds with 4.266 retention time (RT). Top image is the experimental spectra, bottom image was acquired from the NIST library and middle image is a combination of both library and experimental spectra.

Supplementary Fig. 26. m/z spectra of GC-MS data from Milli-Q water (H2O) conditioned with 15:1 PDMS chips fabricated from SU-8 (SU-8) molds with 4.149 retention time (RT). Top image is the experimental spectra, bottom image was acquired from the NIST library and middle image is a combination of both library and experimental spectra.

Supplementary Fig. 27. m/z spectra of GC-MS data from Milli-Q water (H2O) conditioned with 20:1 PDMS chips fabricated from SU-8 (SU-8) molds with 13.457 retention time (RT). Top image is the experimental spectra, bottom image was acquired from the NIST library and middle image is a combination of both library and experimental spectra.

Supplementary Fig. 28. m/z spectra of GC-MS data from Milli-Q water (H2O) conditioned with 20:1 PDMS chips fabricated from SU-8 (SU-8) molds with 6.014 retention time (RT). Top image is the experimental spectra, bottom image was acquired from the NIST library and middle image is a combination of both library and experimental spectra.

Supplementary Fig. 29. m/z spectra of GC-MS data from Milli-Q water (H2O) conditioned with 15:1 PDMS chips fabricated from SU-8 (SU-8) molds with 4.449 retention time (RT). Top image is the experimental spectra, bottom image was acquired from the NIST library and middle image is a combination of both library and experimental spectra.

******Supplementary Fig. 30. m/z spectra of GC-MS data from Milli-Q water (H2O) conditioned with 20:1 PDMS chips fabricated from SU-8 (SU-8) molds with 4.366 retention time (RT). Top image is the experimental spectra, bottom image was acquired from the NIST library and middle image is a combination of both library and experimental spectra.


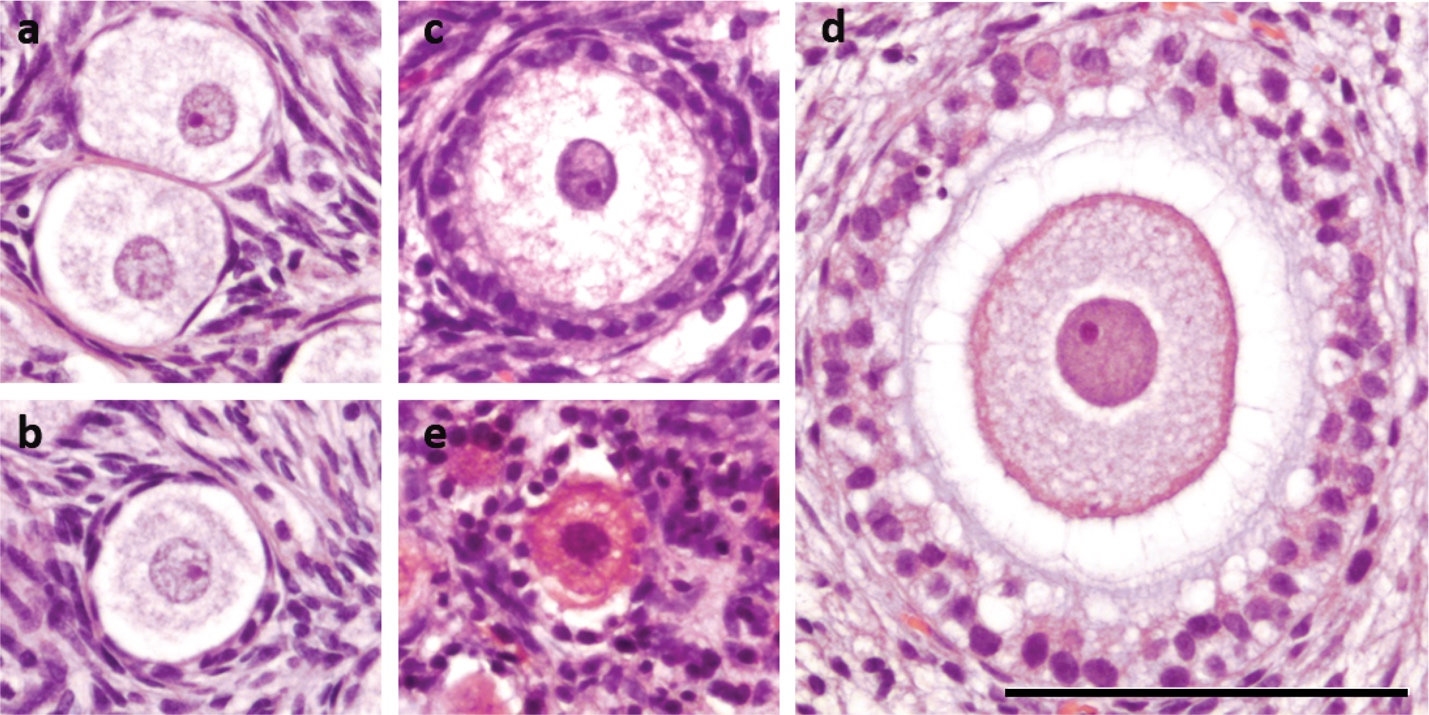


Supplementary Fig. 31. Representative images of domestic cat ovarian follicle stages, with (a) primordial follicles, (b) transitional follicle, (c) primary stage follicle, (d) secondary stage follicle), and (e) an atretic follicle. Scale bar represents 100 µm for all images.

Supplementary Table 1. Putatively identified compounds (based on queries against NIST EI database) of GC-MS analyzed post-wash samples from PDMS chips (15:1) fabricated from 3D-printed (3D) molds with their retention time (RT) and similarity score (SI).

| **Mold** | **Sample** | **RT (min)** | **SI** | **Compound** | **Origin** | **m/z spectra** |
| --- | --- | --- | --- | --- | --- | --- |
| 3D | 15:1 | 9.560  8.911  6.730  4.615; 4.699 | 678  705  870  664; 742 | 2-Methyl-6-phenylpyrazolo[3,4-d][1,3]thiazolo[3,2-a]pyrimidin-4(2H)-one  Methyl N-hydroxybenzenecarboximidoate  Dihydroxydimethylsilane  Hexamethylcyclotrisiloxane | Unknown*  Unknown*  PDMS oligomer  PDMS oligomer | Supp. Fig 32  Supp. Fig 33  Supp. Fig 34  Supp. Fig 35 |

* indicates compounds that were also present in the analyzed control water

Supplementary Fig. 32. m/z spectra of GC-MS data from post-wash samples of PDMS chips fabricated from 3D printed (3D) molds with 9.560 retention time (RT). Top image is the experimental spectra, bottom image was acquired from the NIST library and middle image is a combination of both library and experimental spectra.

Supplementary Fig. 33. m/z spectra of GC-MS data from post-wash samples of PDMS chips fabricated from 3D printed (3D) molds with 8.911 retention time (RT). Top image is the experimental spectra, bottom image was acquired from the NIST library and middle image is a combination of both library and experimental spectra.

Supplementary Fig. 34. m/z spectra of GC-MS data from post-wash samples of PDMS chips fabricated from 3D printed (3D) molds with 6.730 retention time (RT). Top image is the experimental spectra, bottom image was acquired from the NIST library and middle image is a combination of both library and experimental spectra.

Supplementary Fig. 35. m/z spectra of GC-MS data from post-wash samples of PDMS chips fabricated from 3D printed (3D) molds with 4.699 retention time (RT). Top image is the experimental spectra, bottom image was acquired from the NIST library and middle image is a combination of both library and experimental spectra.

Supplementary Fig. 36. Schematic representation of the cell (a) and the tissue (b) culture microfluidics devices.
